# The Enduring Violin

**Published:** 1883-08

**Authors:** 


### THE ENDURING VIOLIN.

Of all the musical instruments the violin is most enduring. Pianos wear out; wind instruments get battered and old-fashioned. All kinds of novelties are introduced into flutes, but the sturdy violin stands on its own merits. Age and use only improve it, and instead of new ones commanding the highest prices, as in the case with other instruments, it is the violin of the few Italian makers of the last three centuries that commands the fabulous prices. It is impossible to handle an old violin without a feeling of veneration, when one reflects on the number of people who have probably played on it, the weary hours it has beguiled, the source of enjoyment it has been, and how well it has been loved.

